# Ongoing seasonal intestinal inflammation in birch pollen allergic patients without gastrointestinal symptoms

**DOI:** 10.1186/2045-7022-1-S1-O15

**Published:** 2011-08-12

**Authors:** Georgios Rentzos, Ulf Bengtsson, Vanja Lundberg, PO Stotzer, Teet Pullerits, Marianne van Hage, Staffan Ahlstedt, Esbjörn Telemo

**Affiliations:** 1 Sahlgrenska University Hospital, Gothenburg, Sweden, Dept. of Respiratory Medicine and Allergology, Section of Allergology, Gothenburg, Sweden; 2 Sahlgrenska Academy, University of Gothenburg, Sweden, Dept. of Respiratory Medicine and Allergology, Section of Allergology, Gothenburg, Sweden; 3 Sahlgrenska Academy, University of Gothenburg, Sweden, Dept. of Rheumatology and Inflammation research, Gothenburg, Sweden; 4 Sahlgrenska University Hospital, Gothenburg, Sweden, Dept. of Internal Medicine, Section of Gastroenterology, Gothenburg, Sweden; 5 Karolinska University Hospital Solna, Dept. of Medicine Clinical Immunology and Allergy Unit, Stockholm, Sweden; 6 Karolinska Institute, Institute of Enviromental Medicine, Experimental Asthma and Allergy Research, Stockholm, Sweden

## Background

A previous study by our group (JACI 2003;112:45-50) showed a significant allergic gastrointestinal inflammation in birch pollen allergic patients with gastrointestinal symptoms during the birch pollen season. The pathophysiological mechanisms of this type of allergic reaction are still poorly studied. However it is not yet explored if all birch pollen patients have signs of gastrointestinal inflammation during the pollen season.

## Aim

To study the immune pathology of the duodenal mucosa in birch pollen allergic patients without gastrointestinal symptoms, outside and during the birch pollen season.

## Methods

All patients were clinically diagnosed as birch pollen allergic. Seven patients allergic to birch pollen without any gastrointestinal symptoms and seven completely healthy individuals were included in the study for comparison. All patients were examined with a standard skin prick test panel and blood samples were analysed for IgE antibodies by component micro-arrays (ISAC). The two groups of patients underwent gastroscopy and duodenal biopsies were taken both during (May-June) and outside the pollen season (November-January) for each patient. Duodenal biopsies were examined with immunostaining for mast cells (IgE and tryptase), eosinophils (eosinophil peroxidase), T-cells (CD3), and dendritic cells (DC) (CD11c).

## Results

In the pollen allergic group there was a significant increase in eosinophils, mast cells and DC’s but not T cells during the pollen season as compared with values outside the season. Interestingly, in the healthy control group we observed a significant increase in CD3+ cells during the birch pollen season, but no change in the other cell types during the pollen season.

## Conclusions

Patients allergic to birch pollen have, regardless of subjective gastrointestinal symptoms, clear signs of an ongoing allergic inflammation in their intestinal mucosa during the pollen season.

